# Supplementary material for: Cell–Fibronectin Interactions and Actomyosin Contractility Regulate the Segmentation Clock and Spatio-Temporal Somite Cleft Formation during Chick Embryo Somitogenesis
Source: Cells. 2022 Jun 22;11(13):2003. doi: 10.3390/cells11132003 (PMC9266262; doi:10.3390/cells11132003)
Supplement: Supplementary file 1 [file cells-11-02003-s001.zip › cells-1769568_Supplementary_Materials_Final/cells-1769568_SupplementaryFigures_Revised PGA.pdf]

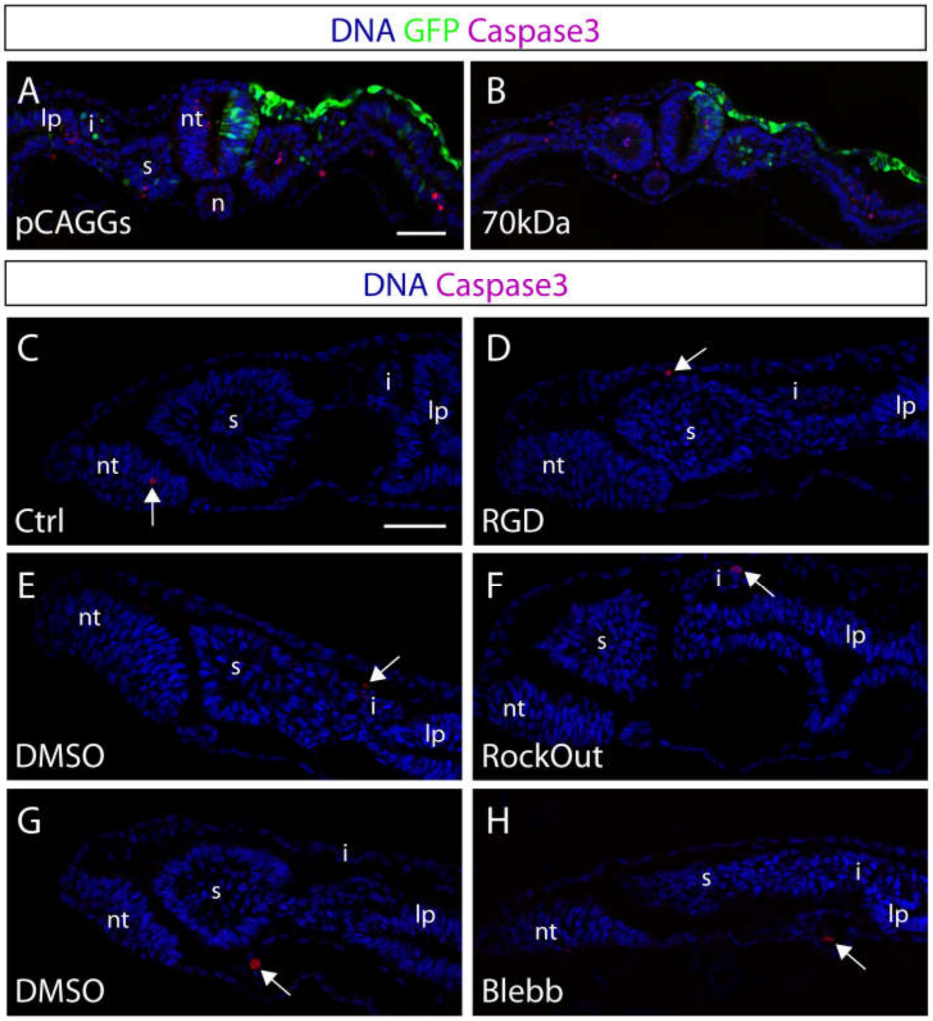

Figure S1. Apoptosis is not enhanced in the experimental culture conditions used.

Immunostaining for activated Caspase3 in transverse sections of **(A,B)** pCAGGs- and 70kDa-electroporated embryos, control and contralateral explants treated with **(C,D)** RGD, **(E,F)** RockOut or **(G,H)** Blebbistatin. Arrows indicate Caspase3-positive cells. DNA (blue); activated Caspase3 (magenta); GFP (green). nt – neural tube; s – somite; i – intermediate mesoderm; lp – lateral plate mesoderm; Blebb – Blebbistatin. Dorsal is on top. Scale bars: 50 μm.

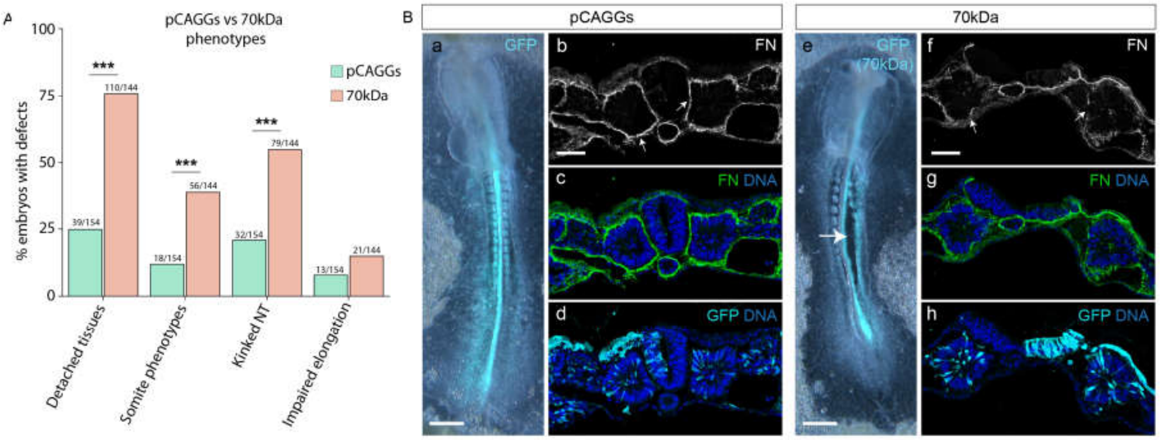

Figure S2. Embryos electroporated with the 70 kDa construct exhibit numerous morphological defects.

(A) Percentage of pCAGGS- and 70kDa-electroporated embryos with morphological defects. **(B)** Representative images of the morphology of embryos electroporated with either (a) pCAGGs or (e) 70kDa (severe phenotype). Transverse sections of (b-d) pCAGGs- and (f-h) 70kDa-electroporared embryos (b-c, f-g) immunostained for fibronectin (FN), (c-d, g-h) DNA, and (d, h) GFP. Electroporated side is on left (a-d) or right (e-h). Detachment of tissues is visible in (e) 70kDa-electroporated embryos (arrow), accompanied by severe disruption in the fibronectin matrix (compare b-c with f-g, arrows). Somite phenotypes include: fewer number, somites only on one side, irregularly shaped, fused, misaligned and diffuse somites. p values were calculated using a chi-square test. ***$p<0.001$. Scale bars: (a, e) 500 μm, (b-d, f-h) 50 μm.

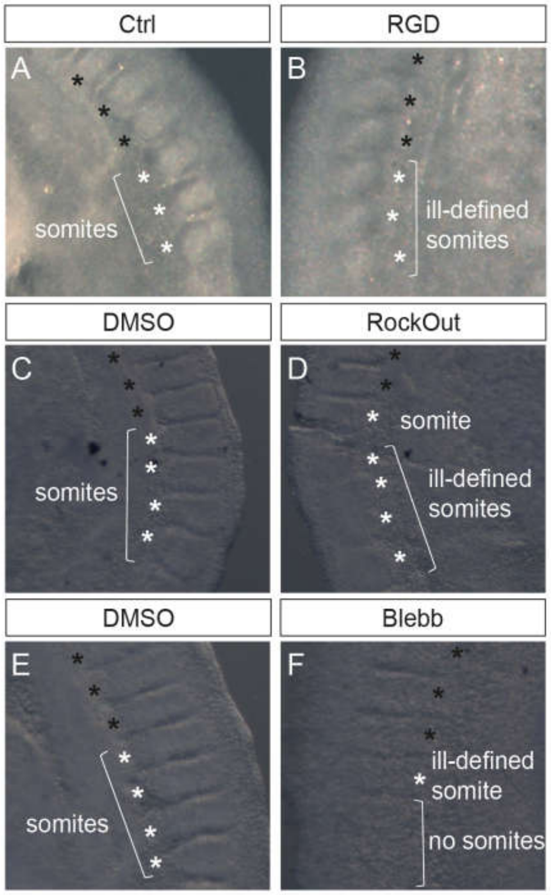

Figure S3. Explants cultured with RGD, RockOut or Blebbistatin form ill-defined somitic structures. Contralateral explant pairs cultured in control and RGD-supplemented medium (**A,B**), DMSO or RockOut-supplemented medium (**C,D**) and with DMSO or Blebbistatin (**E,F**). Dorsal view. Rostral to the top. Black asterisks mark somites formed before culture, while white asterisks indicate somitic structures formed during culture. Somites formed in control conditions (**A,C,E**) are regularly shaped and have clearly demarcated clefts. In contrast, the majority of the somites formed in the presence of RGD or RockOut (**B,D**) have a diffuse appearance, with poorly defined clefts (ill-defined somites). Explants incubated with Blebbistatin form 1-2 ill-defined somites, while no somitic clefts are detected more caudally (**F**).

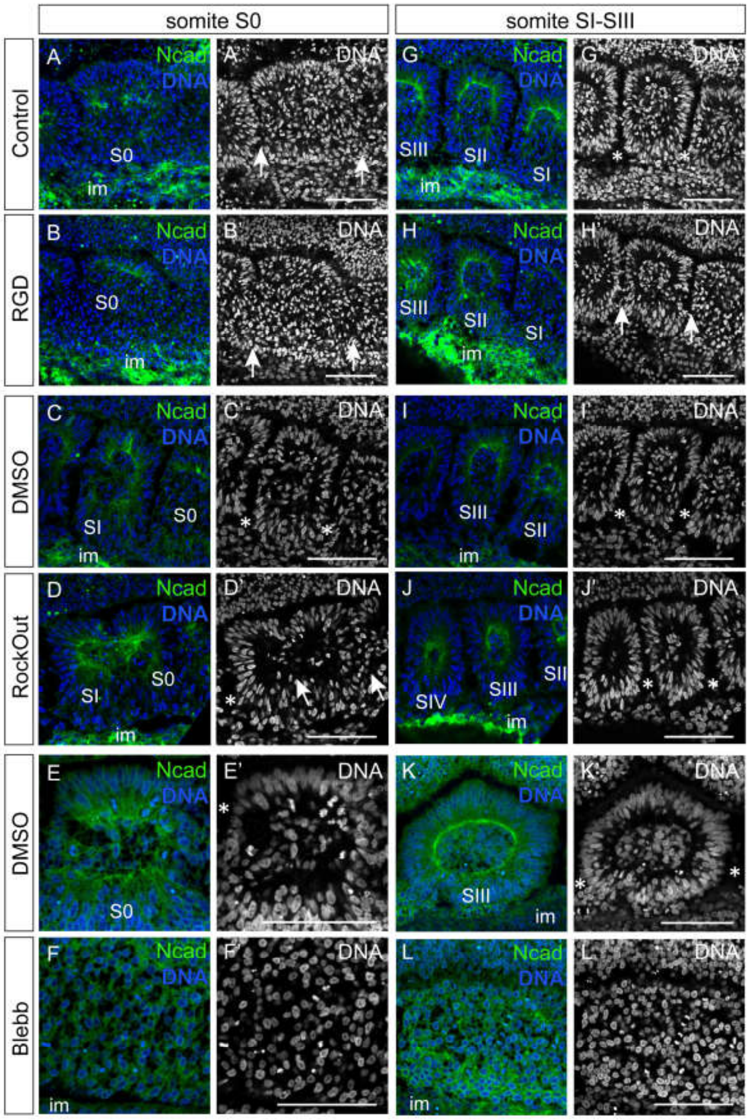

Figure S4. Somite morphology and N-cadherin polarization in paraxial mesoderm exposed to RGD, RockOut or Blebbistatin. Longitudinal views of explant pairs at the level of **(A-F′)** S0 and **(G-L′)** SII/SIII with immunostaining for N-cadherin and staining for DNA. Complete clefts are marked with asterisks and incomplete clefts with arrows. Rostral is to the left, neural tube is on top and intermediate mesoderm (im) on the bottom. In RGD-treated explants N-cadherin polarization in S0 is less advanced, relatively to the contralateral control (**A**,**B**). The S0 of the control explant displays N-cadherin polarization medially, caudally, and laterally (**A**) whereas the S0 of the RGD-treated explant only exhibits N-cadherin polarization medially (**B**). Cleft formation in RGD-treated explants is delayed (arrows, **A′**,**B′**). N-cadherin polarization in S0 and SI of RockOut treated explants was similar to the contralateral control side (**C**,**D**). However, cleft formation was incomplete resulting in fused somites (**D′**). Blebbistatin-treated explants show no N-cadherin polarization (**F**). At the level of SII/SIII, N-cadherin polarization in RGD- and RockOut-treated explants is very similar to that of the contralateral control explants (**G-H**,**I-J**). Cleft formation is less complete in the RGD treated explant compared to the control (**G′**,**H′**), but the RockOut-treated explant is indistinguishable from its control (**I′**,**J′**). At the level of SII/SIII N-cadherin polarization did not occur in the presence of Blebbistatin (**K**,**L**) and no sign of cleft formation was detected (**K′**,**L′**). Blebb – Blebbistatin. Ncad – cadherin. Scale bars: 50 μm.

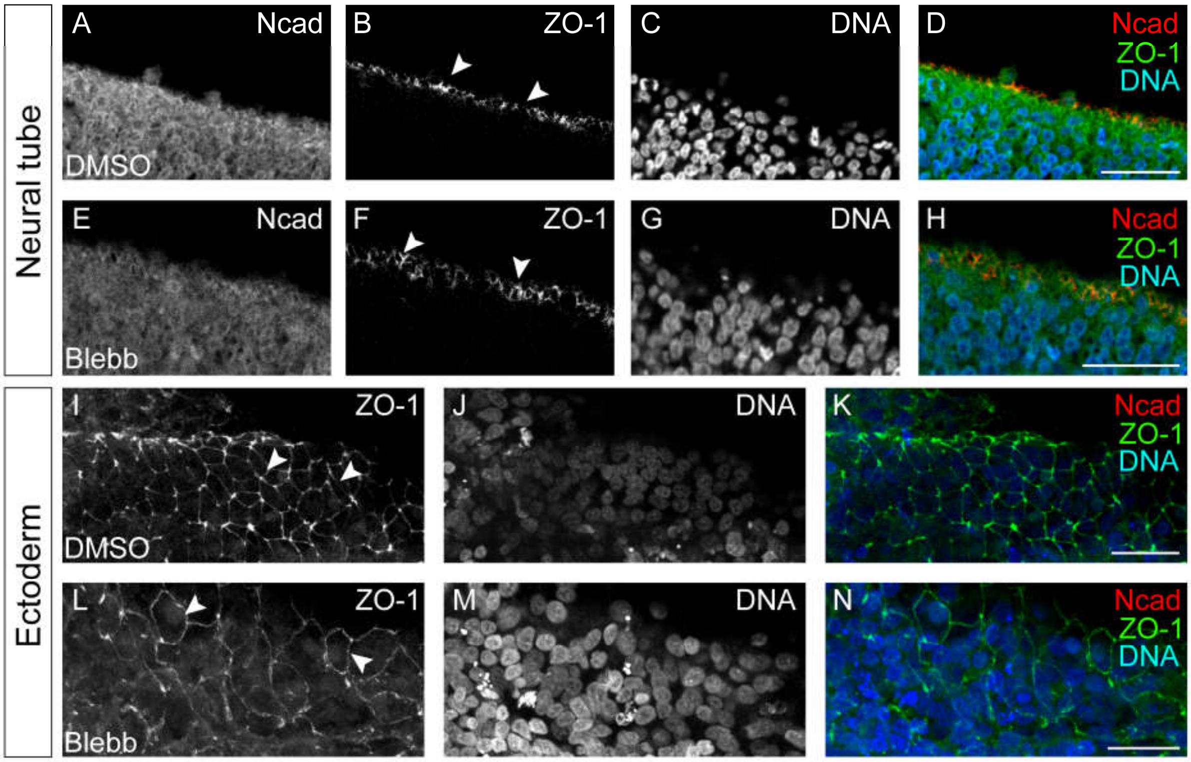

Figure S5. Epithelial structure of the neural tube and ectoderm are unaffected by NM II inhibition.

Control explants **(A-D, I-K)** show apically located ZO-1 labeling (**B, I**, arrowheads) in the neural tube (**A-D**; transverse optical section, medial on top) and in the overlying surface ectoderm (**I-K**; dorsal view of ectoderm). In the presence of Blebbistatin **(E-H, L-N)**, ZO-1 labeling is also present in the apical end of neural tube cells (**F**, arrowheads) and surface ectoderm cells (**L**, arrowheads). Blebb – Blebbistatin. Ncad – N-cadherin. ZO-1 – *Zonula occludens* protein 1. Arrowheads point to ZO-1 labeling. Scale bars: 20 µm.

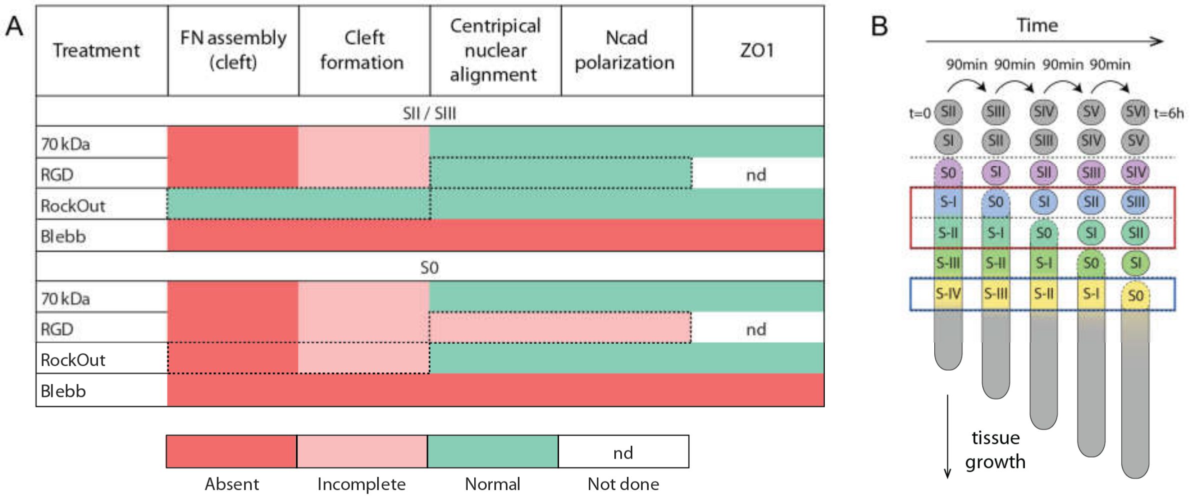

Figure S6. Summary of phenotypes observed in 70kDa-electroporated embryos and explant cultures.

(A) Summary of phenotypes observed at the level of SII/SIII and S0 in embryos electroporated with the 70kDa construct at HH4 and cultured for 26 hours and half explants cultured in the presence of RGD, RockOut or Blebbistatin (Blebb) for 6 hours. Dashed lines indicate changes in the phenotype between S0 and SII/SIII. **(B)** Timeline of exposure of half explants cultured for 6 hours. Red box illustrates that SII/SIII somites at the end of culture were at stage S-II/S-I when the treatments began. Blue box illustrates that S0 at the end of culture was at stage S-IV when the treatments were initiated.
