# Supplementary material for: Cell–Fibronectin Interactions and Actomyosin Contractility Regulate the Segmentation Clock and Spatio-Temporal Somite Cleft Formation during Chick Embryo Somitogenesis
Source: Cells. 2022 Jun 22;11(13):2003. doi: 10.3390/cells11132003 (PMC9266262; doi:10.3390/cells11132003)
Supplement: Supplementary file 1 [file cells-11-02003-s001.zip › cells-1769568_Supplementary_Materials_Final/cells-1769568_Table S1_Revised.pdf]

Table S1: Data from the analysis of *hairy1* expression relating to the experiments of Figure 7. Data from the analysis of explant pairs cultured for the designated times. Columns represent replicates for each of the periods tested and each treatment. diff – different.; nd – not done.

**Number of *hairy1* expression stripes formed during culture**							**Average**
**6 hours + 0 minutes**							
DMSO	4	4	4	nd	nd	nd	**4.0**
DMSO + 0 min	4	4	4	nd	nd	nd	**4.0**
Blebb	3	3	3	3	nd	nd	**3.0**
Blebb + 0 min	3	3	3	3	nd	nd	**3.0**
**6 hours + 60 minutes**							
DMSO	4	4	4	4	4	nd	**4.0**
DMSO + 60 min	5	5	5	4	4	nd	**4.6**
Blebb	3	3	3	3	2	nd	**2.8**
Blebb +6 0 min	4	3	3	3	3	nd	**3.2**
**6 hours + 90 minutes**							
DMSO	4	4	4	4	5	nd	**4.2**
DMSO + 90 min	5	5	5	5	6	nd	**5.2**
Blebb	3	2	3	4	nd	nd	**3.0**
Blebb + 90 min	3	3	4	4	nd	nd	**3.5**
**6 hours + 120 minutes**							
DMSO	4	4	4	nd	nd	nd	**4.0**
DMSO +120 min	5	5	5	nd	nd	nd	**5.0**
Blebb	3	3	3	3	3	3	**3.0**
Blebb + 120 min	4	4	4	4	4	3	**3.8**

**Number of somitic structures formed in culture***							**Average**
**6 hours + 0 minutes**							
DMSO	4	4	4	nd	nd	nd	**4.0**
DMSO + 0 min	4	4	4	nd	nd	nd	**4.0**
Blebb	1	1	0	0	nd	nd	**0.5**
Blebb + 0 min	1	1	1	1	nd	nd	**1.0**
**6 hours + 60 minutes**							
DMSO	4	4	4	4	4	nd	**4.0**
DMSO + 60 min	5	5	5	4	4	nd	**4.6**
Blebb	2	1	0	1	1	nd	**1.0**
Blebb +6 0 min	1	2	0	2	1	nd	**1.2**
**6 hours + 90 minutes**							
DMSO	4	4	4	4	5	nd	**4.2**
DMSO + 90 min	5	5	5	5	6	nd	**5.2**
Blebb	1	0	0	0	nd	nd	**0.3**
Blebb + 90 min	1	1	0	0	nd	nd	**0.5**
**6 hours + 120 minutes**							
DMSO	4	4	4	nd	nd	nd	**4.0**
DMSO +120 min	5	5	5	nd	nd	nd	**5.0**
Blebb	0	1	1	0	1	1	**0.7**
Blebb + 120 min	1	1	1	0	1	1	**0.8**

*Somitic structures in Blebbistatin-cultured explants are always ill-defined.

Hairy1 expression pattern of the two explant pairs after culture							Conclusion
**6 hours + 0 minutes**							
DMSO<br>DMSO + 0 min	Same	Same	Same	nd	nd	nd	**100% same**
Blebb<br>Blebb + 0 min	Same	Same	Same	Same	nd	nd	**100% same**
**6 hours + 60 minutes**							
DMSO<br>DMSO + 60 min	Different	Different	Different	Different	Different	nd	**100% diff**
Blebb<br>Blebb + 60 min	Different	Different	Different	Different	Different	nd	**100% diff**
**6 hours + 90 minutes**							
DMSO<br>DMSO + 90 min	Same	Same	Same	Same	Same	nd	**100% same**
Blebb<br>Blebb + 90 min	Different	Different	Different	Different	nd	nd	**100% diff**
**6 hours + 120 minutes**							
DMSO<br>DMSO +120 min	Different	Different	Different	nd	nd	nd	**100% diff**
Blebb<br>Blebb + 120 min	Same	Same	Same	Same	Same	Different	**83% same**
