# Supplementary figures and images for: Cell–Fibronectin Interactions and Actomyosin Contractility Regulate the Segmentation Clock and Spatio-Temporal Somite Cleft Formation during Chick Embryo Somitogenesis
Source: Cells. 2022 Jun 22;11(13):2003. doi: 10.3390/cells11132003 (PMC9266262; doi:10.3390/cells11132003)

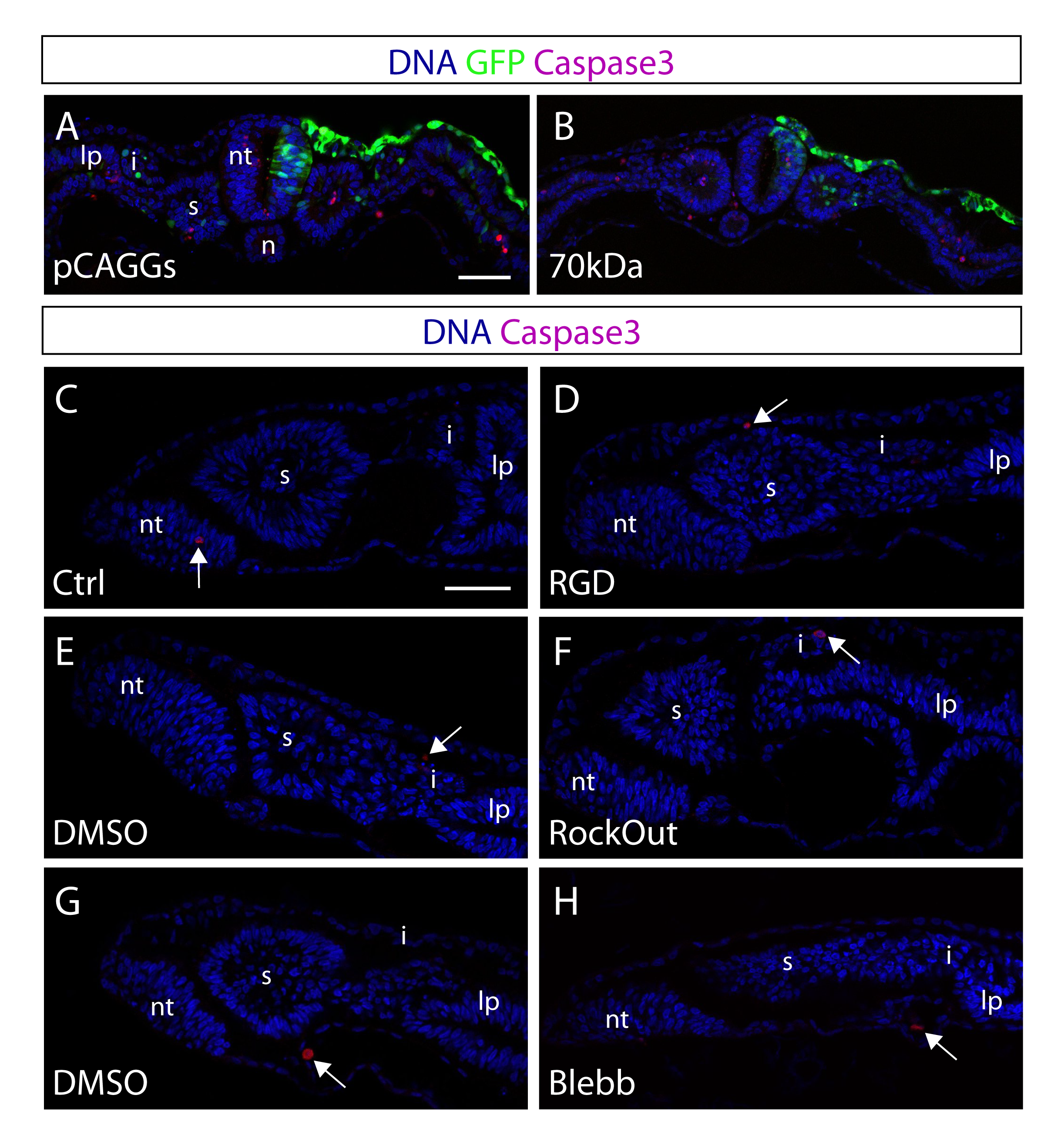

Supplement: Supplementary file 1 [file cells-11-02003-s001.zip › cells-1769568_Supplementary_Materials_Final/Figure_S1.tif]

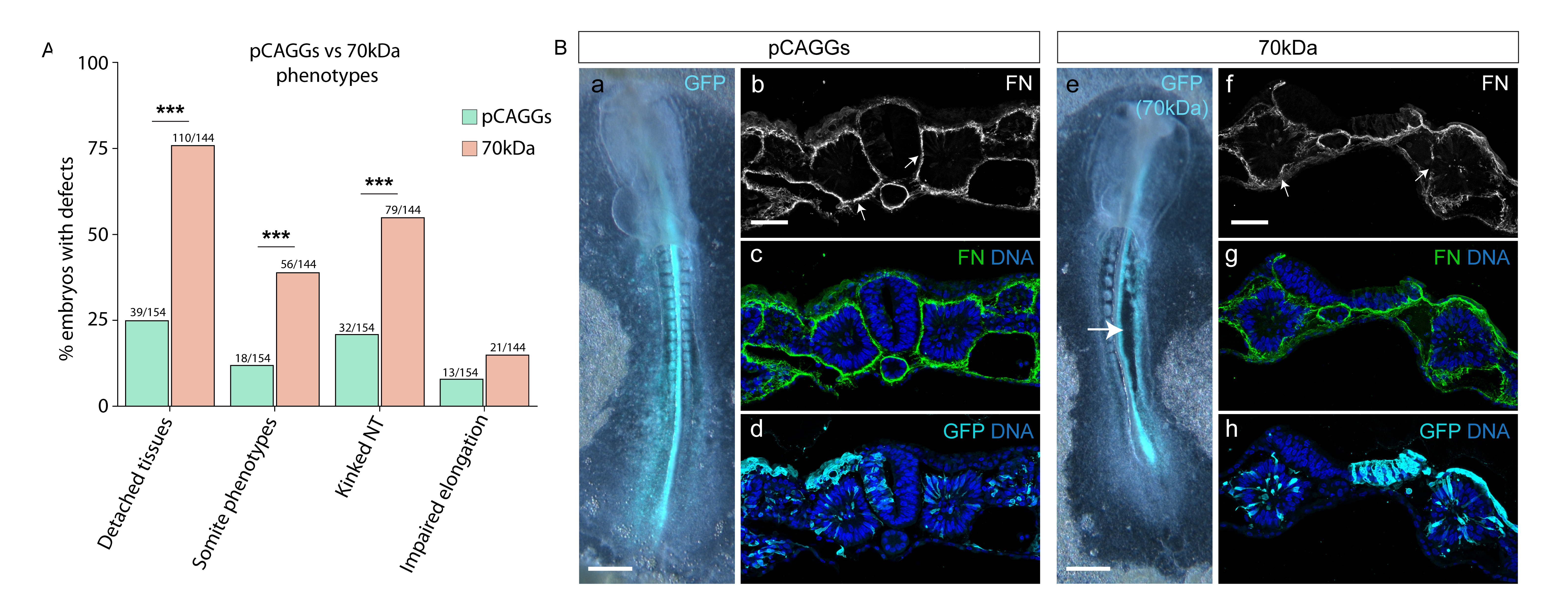

Supplement: Supplementary file 1 [file cells-11-02003-s001.zip › cells-1769568_Supplementary_Materials_Final/Figure_S2.tif]

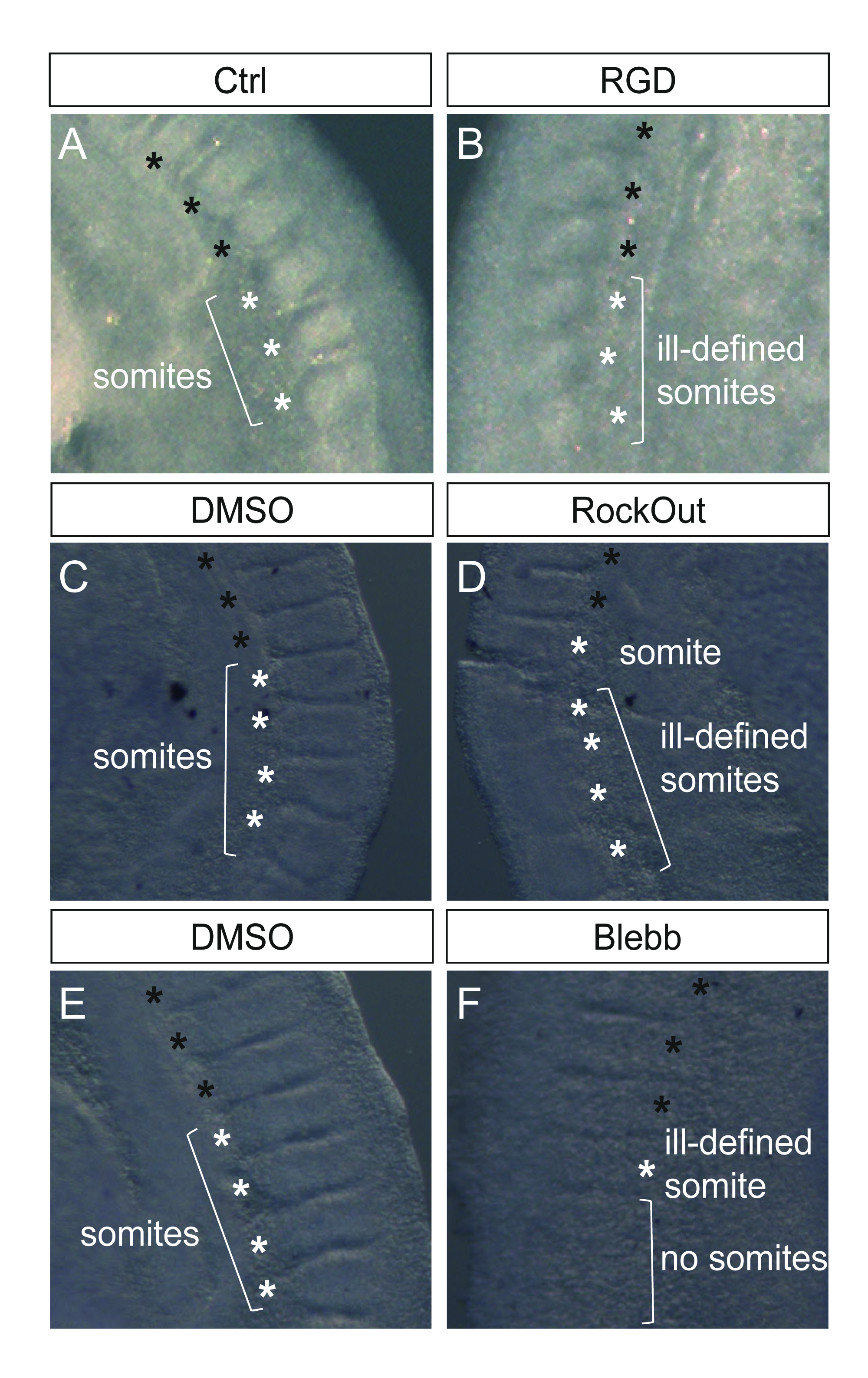

Supplement: Supplementary file 1 [file cells-11-02003-s001.zip › cells-1769568_Supplementary_Materials_Final/Figure_S3.tif]

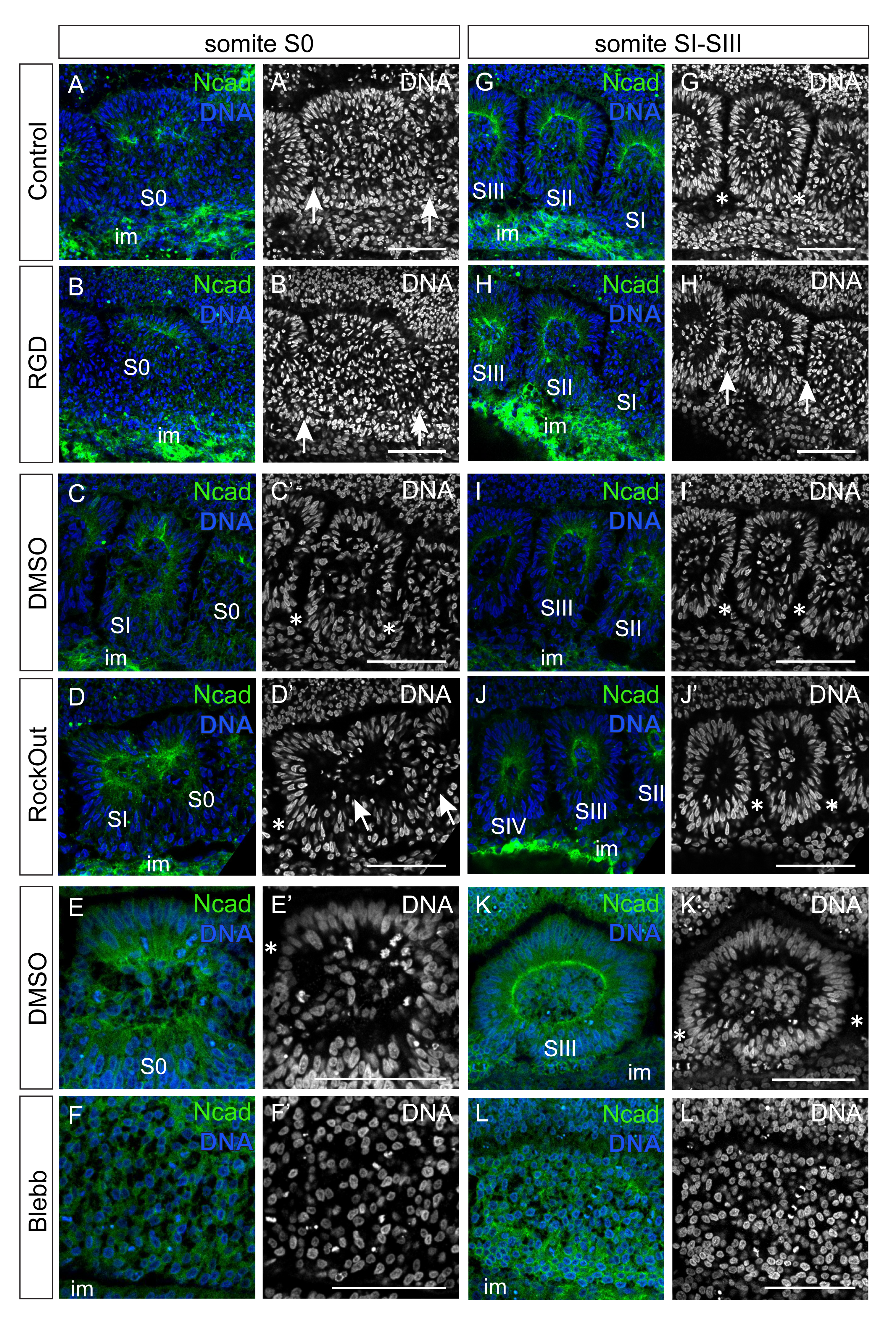

Supplement: Supplementary file 1 [file cells-11-02003-s001.zip › cells-1769568_Supplementary_Materials_Final/Figure_S4.tif]

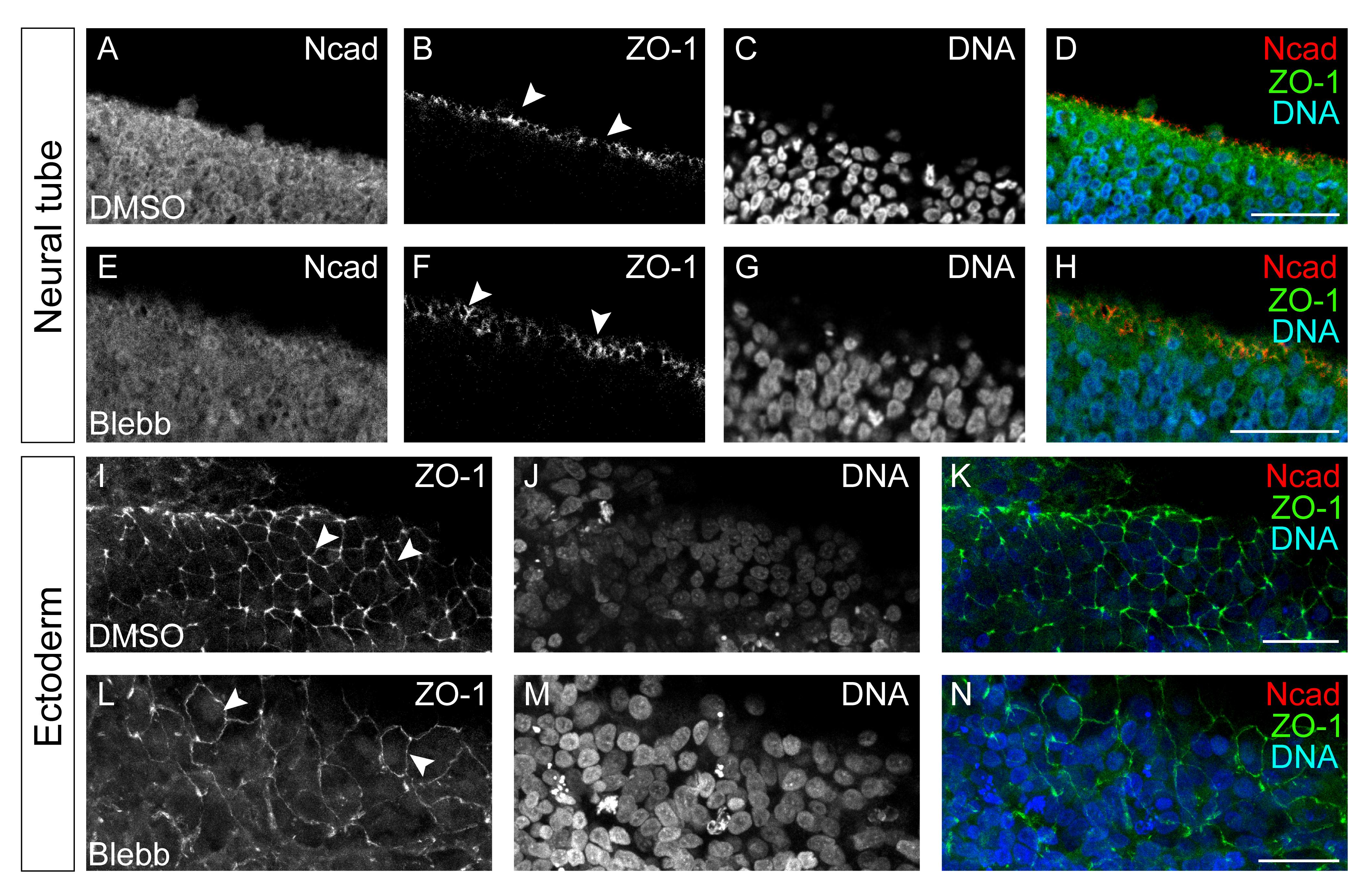

Supplement: Supplementary file 1 [file cells-11-02003-s001.zip › cells-1769568_Supplementary_Materials_Final/Figure_S5.tif]

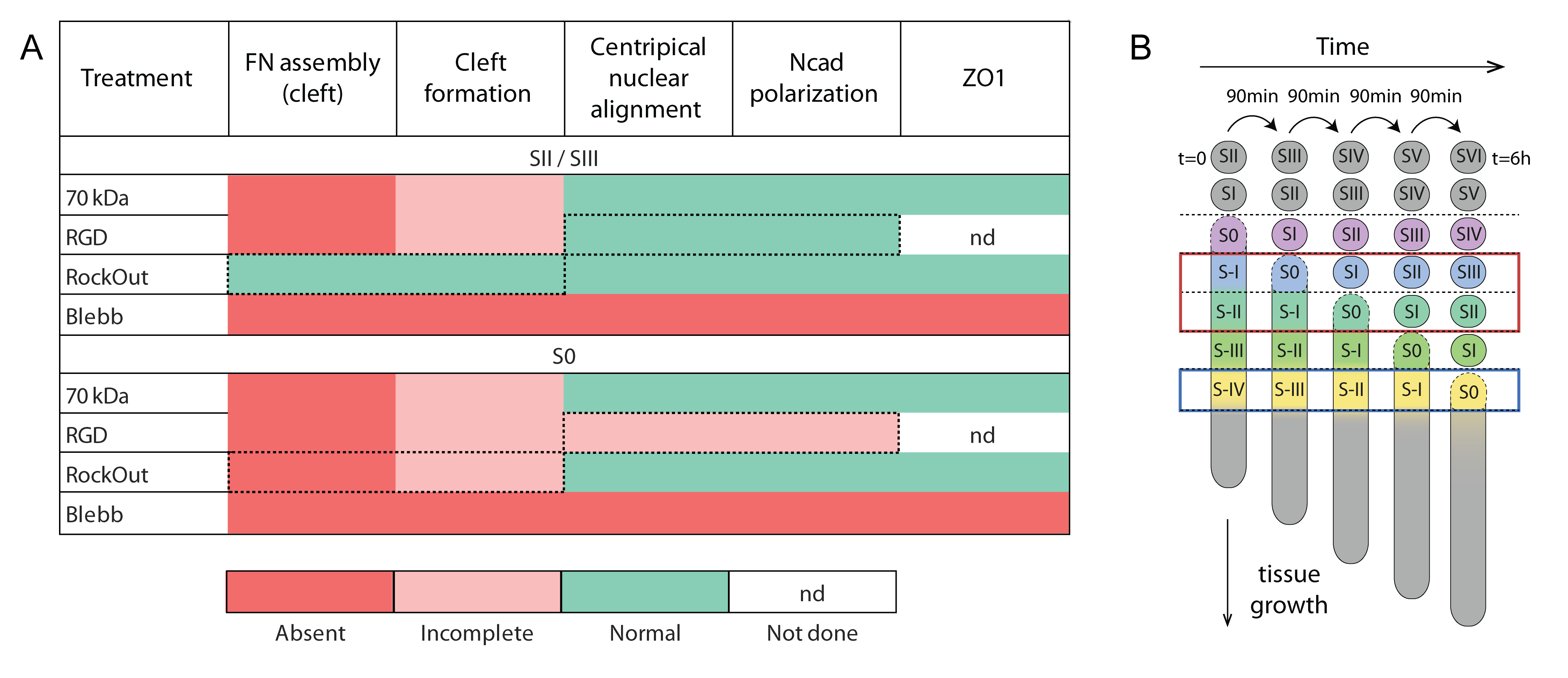

Supplement: Supplementary file 1 [file cells-11-02003-s001.zip › cells-1769568_Supplementary_Materials_Final/Figure_S6.tif]
